# Differentiation between non-neural and neural contributors to ankle joint stiffness in cerebral palsy

**DOI:** 10.1186/1743-0003-10-81

**Published:** 2013-07-23

**Authors:** Karin L de Gooijer-van de Groep, Erwin de Vlugt, Jurriaan H de Groot, Hélène CM van der Heijden-Maessen, Dennis HM Wielheesen, Rietje (M) S van Wijlen-Hempel, J Hans Arendzen, Carel GM Meskers

**Affiliations:** 1 Department of Rehabilitation Medicine, Leiden University Medical Centre, Leiden, the Netherlands; 2 Department of Mechanical Engineering, Delft University of Technology, Delft, the Netherlands; 3 Rijnland’s Rehabilitation Centre, Leiden, the Netherlands; 4 Sophia Rehabilitation, Gouda, the Netherlands; 5 Dubai Bone and Joint Centre, Dubai, UAE

**Keywords:** Cerebral palsy, Movement disorder, Ankle joint, Stiffness, Spasticity, Contracture, Neuromechanics, System identification, Neuromuscular modeling

## Abstract

**Background:**

Spastic paresis in cerebral palsy (CP) is characterized by increased joint stiffness that may be of neural origin, i.e. improper muscle activation caused by e.g. hyperreflexia or non-neural origin, i.e. altered tissue viscoelastic properties (clinically: “spasticity” vs. “contracture”). Differentiation between these components is hard to achieve by common manual tests. We applied an assessment instrument to obtain quantitative measures of neural and non-neural contributions to ankle joint stiffness in CP.

**Methods:**

Twenty-three adolescents with CP and eleven healthy subjects were seated with their foot fixated to an electrically powered single axis footplate. Passive ramp-and-hold rotations were applied over full ankle range of motion (RoM) at low and high velocities. Subject specific tissue stiffness, viscosity and reflexive torque were estimated from ankle angle, torque and triceps surae EMG activity using a neuromuscular model.

**Results:**

In CP, triceps surae reflexive torque was on average 5.7 times larger (*p =* .002) and tissue stiffness 2.1 times larger (*p =* .018) compared to controls. High tissue stiffness was associated with reduced RoM (*p <* .001). Ratio between neural and non-neural contributors varied substantially within adolescents with CP. Significant associations of SPAT (spasticity test) score with both tissue stiffness and reflexive torque show agreement with clinical phenotype.

**Conclusions:**

Using an instrumented and model based approach, increased joint stiffness in CP could be mainly attributed to higher reflexive torque compared to control subjects. Ratios between contributors varied substantially within adolescents with CP. Quantitative differentiation of neural and non-neural stiffness contributors in CP allows for assessment of individual patient characteristics and tailoring of therapy.

## Background

Cerebral palsy (CP) comprises a variety of non-progressive upper motor neuron (UMN) lesions occurring in the developing fetal or infant brain. The resulting movement and posture disorders are generally characterized by loss of muscle strength, i.e. paresis, improper muscle activation by e.g. increased reflexes and loss of coordination by e.g. flexion synergies. In addition, changes of tissue viscoelastic properties may modulate the characteristics of the primary motor disorders [1,2]. Spastic CP is the most common type of CP [3], which is characterized by increased joint stiffness (resistance to movement). Increased joint stiffness in the relaxed condition can be of either neural (hyperreflexia, “spasticity”) or non-neural origin (altered tissue viscoelastic properties “contracture”) [4]. Treatment of spastic CP is generally aimed at diminishment of joint stiffness in order to improve passive and active joint range of motion. In case of suspected neural origin, therapy is aimed at reducing muscle activation and blocking the stretch reflex loop by botulinum toxin [5], intra thecal baclofen [6] or selective dorsal rhizotomy [7]. In case of suspected non-neural origin, i.e. changes in viscoelastic properties of muscle and connective tissues, corrective casting, splinting and surgical lengthening can be applied [8]. Current manual tests, like the Ashworth [9] and Tardieu [10], are based on the paradigm of increased reflex activity as a result of neural damage, leading to a velocity dependent joint resistance or spasticity [11]. This paradigm is however an oversimplification [4]. Inherently, by manual testing, it is not possible to quantitatively discriminate between underlying neural and non-neural contributors to joint stiffness as each of these contributors may generate a velocity dependent joint resistance. This makes the selection of treatment aiming at the dominant contributor difficult.

De Vlugt et al. [12] developed an instrumented method to quantify neural and non-neural contributors to joint stiffness for the ankle joint in patients with chronic stroke. The ankle was rotated in a precise and controlled way using a robotic manipulator. Using neuromuscular modeling, the key neural and non-neural contributors to ankle joint stiffness were quantified from recorded ankle torque and EMG of leg (below the knee) muscles. Compared to healthy subjects, patients with stroke showed increased tissue stiffness and to a lesser extent increased reflex activity.

The objective of the present study was to quantify neural and non-neural contributors to ankle joint stiffness in patients with spastic CP and to assess its validity and reliability. A quantitative discrimination between the neural and non-neural components of joint stiffness in CP gives insight in pathophysiological mechanisms and may provide a strong instrument for development of tailored intervention strategies and their follow-up.

## Methods

### Participants

Twenty-three adolescents with CP (mean age (SD) and range: 14.9 (2.4) y, 12–19 y, fifteen male) were recruited from the outpatient clinics of the Rijnlands Rehabilitation Centre and the Department of Rehabilitation Medicine of the Leiden University Medical Centre, Leiden, the Netherlands. Table 1 provides the patient characteristics. Inclusion criteria comprised diagnosis of spastic CP and a gross motor function [13] (GMFCS) of I, II or III. Patients with a GMFCS of IV were excluded because of possible interference of the outcome with muscle disuse or atrophy. Other exclusion criteria were concomitant neurological diseases, orthopedic problems of the lower extremities, casting, botulinum toxin A injections within the previous 4 months, previous orthopedic surgery or tendon and tissue surgery of the leg, orthopedic surgery of other body parts within the last 12 months, previous selective dorsal rhizotomy or intra thecal baclofen treatment and inability to participate in the tests. Eleven healthy subjects (mean age (SD) and range: 15.1 (2.1) y, 12–18 y, six males), matched for age and sex, were recruited as a control group. Required sample size estimation was based on previous data [12]. The medical ethics committee of the Leiden University Medical Centre approved the study. Written informed consent was obtained from participants.

**Table 1:** Characteristics of study population

	**Cerebral palsy (n = 23)**	**Healthy subjects (n = 11)**
Age, mean (SD)	14.9 (2.4)	15.1 (2.1)
Male gender, n (%)	15 (65)	6 (55)
Unilateral, n *(*%*)*	8 (35)	NA
GMFCS^*^ I, II, III, n (%)	20 (87)	NA
	2 (9)	
	1 (4)	
Ashworth, median (range)	1 (0-2)^***^	NA
SPAT^**^, median (range)	2 (0-3)^***^	NA

^*^GMFCS Gross Motor Function Classification System, ^**^SPAT Spasticity test ^***^, Of one patient, data not available.

### Instrumentation

**Figure 1:**
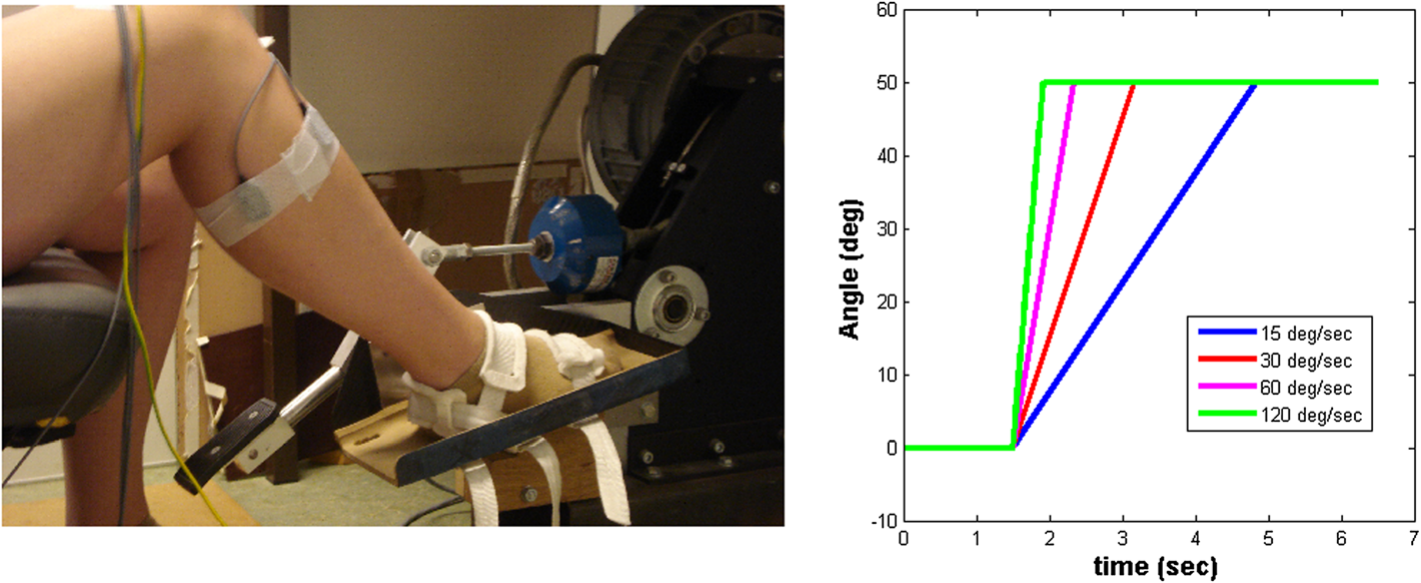
**Measurement set-up (left) and applied ankle joint rotations (right).** Ankle joint rotations were applied at 4 different velocities over the individual range of motion.

Subjects were seated with their foot fixated onto an electrically powered single axis footplate (MOOG FCS Inc., Nieuw Vennep, the Netherlands). Subjects were seated with their hip and knee positioned at approximately 80° and 70° of flexion respectively (Figure 1). The thigh was held in place through the seat support of the chair; movement of the shank in cranial- caudal direction was ensured by careful aligning axis of rotation of the motor with the ankle axis. Movement of the shank in medio-lateral was limited by fixation of the thigh. The ankle was positioned at zero degrees onto the footplate of the manipulator, perpendicular to the leg (neutral position). A positive rotation of the manipulator was defined to equal dorsiflexion of the foot and a negative rotation, plantar flexion of the foot. Range of motion of the manipulator was mechanically constrained to plus and minus 30 degrees with respect to the neutral position. The axis of rotation of the ankle and footplate were aligned by visually minimizing knee translation in the sagittal plane during manual rotation of the footplate. The motor was driven to rotate the ankle by either a torque for the assessment of ankle range of motion (RoM), or by a position to impose ramp-and-hold (RaH) rotational stretches of the triceps surae for the identification of joint stiffness. During movement, flat foot placement was ensured by visual inspection. Muscle activation of the tibialis anterior (TA) and triceps surae muscles (TS: soleus, lateral and medial gastrocnemius) was recorded by surface electrodes (electromyography, EMG), using a Delsys Bagnoli 4 system (Delsys, Boston MA, USA). Inter electrode distance was 10 mm. EMG signals were sampled at 2500 Hz, online band pass filtered (20–450 Hz) and offline rectified and low pass filtered (3th-order Butterworth) at 20 Hz. Reaction torque and ankle rotational angle were recorded at 250 Hz sample rate and low pass filtered at 20 Hz (3th-order Butterworth).

### Protocol

Measurements were performed on the most affected ankle of each patient and at the right ankle in case of controls. Maximum plantar and dorsal flexion angles were assessed by a gradually increasing flexion torque from 0 to a maximum value of 15 Nm. RoM was defined as the difference between the maximum plantar and dorsal flexion angle and used as boundary for the subsequent RaH rotations. During the RaH rotations, the ankle was rotated at 4 different angular velocities (15, 30, 60, 120 deg/sec) over the individually assessed RoM, starting in maximal plantar flexion. RaH rotations were started at random time instants. The hold phase lasted 4 seconds after which the ankle was moved back again to the neutral position. Time to cover a complete RaH rotation did not exceed 15 sec. Rest periods of about 30 sec were introduced between each RaH rotation to avoid hysteresis effects [14]. All RaH rotations were performed twice. Thus, the complete experimental procedure consisted of 1 RoM and 2 times 4 RaH rotations. Subjects were asked to remain relaxed during the entire experiment and not actively resist any motion. EMG prior to RaH rotation was offline checked to be between minus and plus 3 times standard deviation from the lowest EMG value over the whole signal as determined by a moving average procedure (window width 1 sec.). RaH rotations not fulfilling this requirement were discarded from further analysis.

### Model description and validation

To distinguish between the neural and non-neural contributions to ankle joint stiffness, a nonlinear neuromuscular model of the ankle joint was used by which the ankle torque was predicted and matched to the measured ankle torque using EMG and ankle angle as input. The model included a Hill-type muscle model to describe the torque contribution from muscle activation induced by stretch reflexes. Hill-type models account for the effect of muscle length and lengthening velocity on muscle force. Passive torque from viscoelasticity (parallel elastic element) was modeled by exponential force-length and force-velocity functions. Tendon stiffness (series elastic element) was assumed to be infinitely stiff [12]. The full description of the model can be found in de Vlugt et al. [12]. The model was fitted to the measured ankle torque defined within a time frame starting 0.5 sec before the start of the ramp till 0.5 sec after the end of the ramp, which was in the hold phase. Model parameters where estimated for each single RaH rotation by minimizing the quadratic difference (error function) between the measured and predicted ankle torque. The validity of the model was determined for each RaH rotation by the variance accounted for (VAF):

(1) $$\mathrm{VAF}=\left(1-\frac{\sum_{i=1}^{n}{\left(T_{\mathrm{measured},}{}_{i}-T_{\mathrm{mod}\mathrm{el},}{}_{i}\right)}^{2}}{\sum_{i=1}^{n}T_{\mathrm{measured},i}^{2}}\right)*100\%$$

With *i* the sample time and *n* the number of data points used for the parameter estimation. *T*_*measured*,*i*_ is the measured ankle reaction torque and *T*_mod*el*,*i*_ the predicted ankle torque. Those rotations exhibiting a VAF score lower than the mean VAF over all rotations minus 2 times standard deviation were excluded from analysis.

Primary outcome parameters were RoM, tissue stiffness and viscosity and torque from triceps surae (TS) and tibialis anterior (TA) stretch reflexes. As passive tissue stiffness and viscosity strongly depend on joint angle, values at the maximal common dorsal flexion angle of all subjects were calculated for inter-subject analysis. This particular angle was chosen as exhibiting probably the largest contrast between subjects [12]. Model simulations and data analyses were performed in MATLAB (The Mathworks Inc., Natick MA). An extensive validity and reliability analysis of the used method and the estimated model parameters was performed previously [12].

### Statistical analysis

Difference in RoM between patients with CP and healthy controls was tested using an unpaired t-test. A linear mixed model was used to determine the difference in primary outcome variables between healthy controls and patients with CP (random factor) and to assess the effect of velocity (fixed, repeated factor). Stepwise linear regression procedures and one way ANOVA with Bonferroni correction were applied to assess associations of primary outcome variables with RoM and secondary outcome variables i.e. speed of ankle rotation, age, gender, GMFCS [13], Ashworth [9] and spasticity test (SPAT) [15] scores. Inter-trial variability was assessed using intraclass correlation coefficients (ICC, 2-way mixed model). Statistical analysis was performed using SPSS 17.0 (SPSS Inc.) and GraphPad Prism 5 (Graphpad Software) with a significant level of .05.

## Results

One subject (healthy) could not complete the RaH measurement due to insufficient relaxation and in this particular case only the RoM was used. In 2 other healthy subjects 1 RaH rotation had to be excluded due to technical problems. In total, 16 of the 256 RaH rotations from 9 subjects (8 CP), were excluded due to poor model fits (10, 4% of 256 RaH rotations) or insufficient relaxation (6, 2 % of 256 RaH rotations). The VAF of the remaining RaH rotations was above 98.9%, meaning that the model could well describe ankle torque dynamics.

### Range of motion

**Figure 2:**
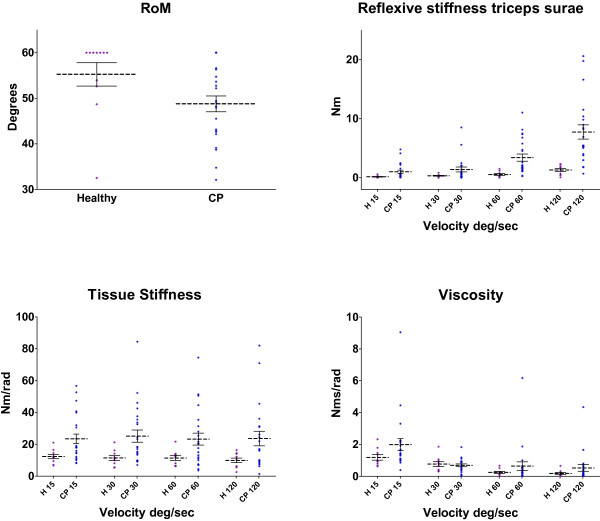
**Primary outcome parameters.** Range of motion (top left), triceps surae reflexive stiffness (top right), tissue stiffness (bottom left) and viscosity (bottom right) for patients with cerebral palsy (CP, blue) and healthy controls (H, purple). The dotted line gives the mean value with the corresponding standard error. Each dot represents an individual result of a subject. Tissue stiffness and viscosity were determined at the same ankle angle for all subjects, which was 2 deg ankle dorsal flexion.

RoM in dorsiflexion was significantly smaller in CP (*t* = 2.10, *p* = .044), see Figure 2 (top left). Of all subjects, 4 patients with CP (12%) and 7 healthy controls (64%) had a RoM of at least 60 deg. The smallest maximal dorsal flexion angle among all subjects was 2 deg.

### Non-neural contributors to joint stiffness: tissue stiffness and viscosity

Tissue stiffness was independent of velocity (*F =* 0.35, *df =* 3, *p =* .79) and was significantly larger in CP compared to healthy controls (*F =* 6.28, *df =* 1, *p =* .018), see Figure 2 (bottom left). There was a large variation in tissue stiffness within the CP group. Viscosity decreased with angular velocity (*F* = 9.86, *df =* 3, *p* < .001). We found no significant difference between the groups regarding ankle viscosity (*F* = 1.35, *df =* 1, *p* = .254).

### Neural contributor to joint stiffness: reflexive torque

TS reflexive torque (Figure 2, top right) was higher in CP than in healthy controls (*F =* 11.6, *df =* 1, *p =* .002) and the difference increased with velocity (*F =* 4.61, *df =* 3, *p =* .009). TS reflexive torque showed a large variation within the group with CP. TA reflexive torque was not significantly different between CP and healthy controls (*F =* 2.864, *df =* 1, *p =* .104) and did not change with velocity (*F =* 0.602, *df =* 3, *p =* .620).

### Relation tissue stiffness and range of motion

**Figure 3:**
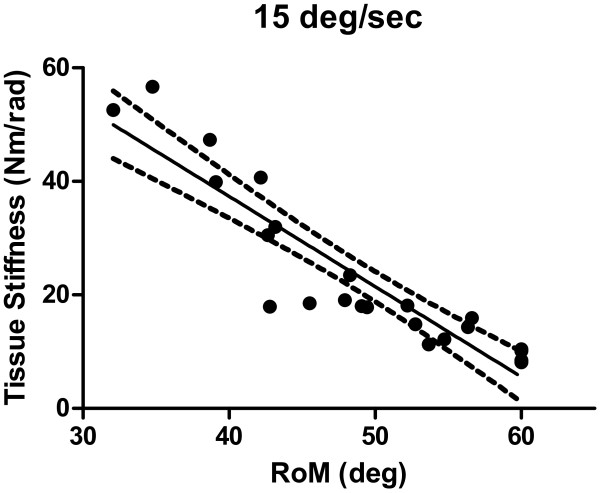
**Relation between tissue stiffness and range of motion for adolescents with cerebral palsy (CP).** Tissue stiffness (15 deg/sec) was determined at 2 deg ankle dorsal flexion. Each dot represents the result of an individual subject. A linear regression line with its 95% confidence interval is fitted through the data. The total explained variance was 84%.

Tissue stiffness at the lowest ankle rotation speed appeared to be the best predictor of ankle RoM (*β = −*0.45 *se* 0.06, *p <* .001, Figure 3) with a total explained variance of 84%.

### Relation tissue stiffness and reflexive torque

For patients with CP, tissue stiffness at the lowest ankle rotation speed (15 deg/sec) was on average 4.2 times higher than reflexive torque at the highest ankle rotation speed (120 deg/sec) with a standard deviation of 3.3 indicating a substantially variation between subjects. Total explained variation of reflexive torque (120 deg/sec) by tissue stiffness (15 deg/sec) was 38%. Association between tissue stiffness and reflexive torque was low (ICC: less than .5).

### Relation with clinical phenotype

**Figure 4:**
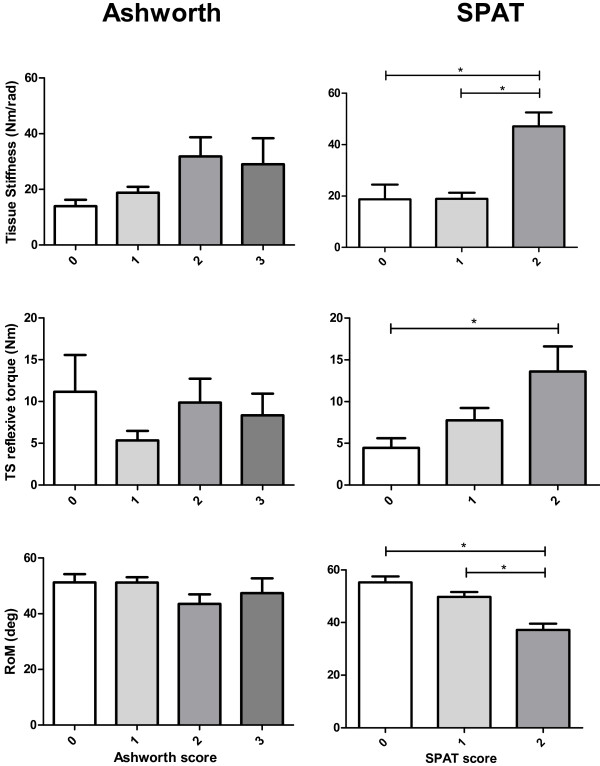
**Relation of outcome parameters with Ashworth and spasticity test (SPAT).** Mean with standard error for tissue stiffness (15 deg/sec), triceps surae (TS) reflexive torque (120 deg/sec) and range of motion (RoM) for groups of patients with different Ashworth and SPAT scores. *significantly different (*p* < .05) by one way ANOVA with a Bonferroni post hoc test.

Using stepwise linear regression SPAT score was the only variable that was significantly associated with tissue stiffness (*β =* 15.8 *se* 4, *p =* .001). For reflexive torque, both SPAT score and age were significant positive contributors (*β =* 3.8 *se* 1.46, *p =* .02 and *β =* 0.88 *se* 0.41, *p =* .049). Tissue stiffness (15 deg/sec, *p* = .002), TS reflexive torque (120 deg/sec, *p* = .032) and RoM (*p = .*001) differed significantly with respect to SPAT but not Ashworth score (Figure 4).

### Reliability

Tissue stiffness showed a good conformity between the two repetitive RaH rotations especially at the lowest ankle rotation speed: ICC .93 at 15 deg/sec. For reflexive torque, inter-trial reliability was especially good at the highest ankle rotation speed: ICC .80 at 120 deg/sec. Reliability was similar for CP and healthy subjects.

## Discussion

Ankle joint stiffness in CP was successfully separated into its neural and non-neural components using an instrumented and model based approach. Compared to healthy subjects, patients with CP showed a smaller RoM, higher TS reflexive torque and higher tissue stiffness. Ratios between contributors varied substantially within the group with CP.

### Higher tissue stiffness and smaller range of motion in cerebral palsy

Previously, in larger groups of children with CP, RoM was associated with level of spasticity as expressed by Ashworth score and GMFSC I-II [16-19]. Decreased RoM in CP is explained by increased passive tissue stiffness [19], likely originating from changes in the mechanical property of fiber bundles and/or fewer sarcomeres (in series) which might result in increased sarcomere length [20-22] or actively, i.e. hypertonia [23]. In vivo measurements in CP show that muscles appeared to undergo much higher stresses with increased muscle length [20] and torque-angle relationships are much steeper in CP [24], which is supported in our study by the correlation between tissue stiffness and RoM (Figure 3). RoM measurements at low speed may therefore represent passive tissue stiffness, i.e. “static” contracture [23]. Note that despite the instruction to the subjects to relax, tissue stiffness may be modulated by a constant level of (increased muscle activation) [25]. Separation of these components requires further effort.

### Clinical implication: variation in tissue stiffness and reflexive torque

Even in this relatively mild affected group of adolescents with CP, a large inter-subject variation was found for the ratio between TS reflexive torque and tissue stiffness. Association between the two was low. This variation is the rationale for pursuing the development of personalized therapy. A variation in CP may be induced by ageing and the corresponding growth spurt in puberty since we measured adolescents in a wide age range (12–19 years). It was suggested previously that the role of passive stiffness may increase over reflex activity with age in children with spastic diplegic CP [26] and that the range of dorsiflexion of the ankle joint in CP decreases on average 19 deg during the first 18 years of life [27]. We found an association of reflex activity but not tissue stiffness with age in the present study. The present study was however not designed to study age effects.

Correlation of SPAT score with both tissue stiffness and reflexive torque underlines the fact that it is difficult to split the neural and non-neural component with the manual tests such as SPAT. In future work we will measure more patients and with a wider range of GMFCS and SPAT scores to study this correlation more extensively.

The present and former [12] studies show that the instrumented approach can be used in different patient groups to quantitatively determine neural and non-neural contributors of ankle joint stiffness.

### Non-neural and neural components in cerebral palsy compared to stroke

TS reflexive torque was more dominant in CP than in stroke [12]. For the patients with an Ashworth score of 1, the ratio between TS reflexive torque at the highest ankle rotation speed and tissue stiffness at the lowest ankle rotation speed was for CP three times higher than for stroke (CP ≈ 0.3 and stroke ≈ 0.1). In contrast to stroke, viscosity was not significantly increased in CP. RoM was smaller in stroke compared to CP, reflecting also the higher tissue stiffness component in the stroke group. CP differs from stroke by onset of the disease with respect to age. The main question is whether the differences between stroke and CP may be explained by purely an age effect or whether there might be etiological differences.

### Reliability and validity

Tissue stiffness and reflexive torque could both be reliably estimated: tissue stiffness especially at the low ankle rotation speed and reflexive torque at the high speeds. This illustrates the feasibility of the method to distinguish contributors to joint stiffness on an individual basis. As expected, reflexive torque and not tissue stiffness was significantly influenced by ankle rotation speed and especially tissue stiffness was associated with RoM at low speed. Significant associations of SPAT score with both tissue stiffness and reflexive torque show agreement with clinical phenotype.

### Limitations

We selected patients with CP with a relatively high GMFSC score (median of 1) to create a homogenous population. This limits validity of the present study and prevents more extensive elaboration of the relation with clinical phenotype which will be important for goal directed therapy. There were only 3 patients with a GMFCS score higher than 1, so correlations of GMFCS with neuromuscular parameters could not be studied well. The present study did not differentiate contributions of gastrocnemius and soleus and was performed at 1 knee angle. Joint stiffness should also be assessed in relation to functional movements, like walking using static and dynamic measurements [28]. In this study we were able to split neural and non-neural contributors to increased joint stiffness. However, the neural component in this study comprised only the reflex activity and not cross bridge dynamics and background muscle activity during passive (and active) conditions. Achilles tendon stiffness was assumed to be of a magnitude greater than the total (active and passive) muscle stiffness for the current conditions applied in this study [29] and was taken in our model as infinitely stiff, considering the low torque and passive conditions [12] applied in the present study.

Future work will comprise the assessment of Achilles tendon stiffness by ultrasound measurements, the assessment of joint stiffness as a function of ankle rotation angle in a more detailed way, measurements at different knee angles and under functional (loaded) conditions.

## Conclusion

Using a novel instrumented assessment technique, patients with CP showed a smaller RoM and higher tissue stiffness and reflexive torque compared to control subjects. Good reliability and validity of the assessment technique combined with considerable intra-individual variance are a base for individual tailored therapy.

## Competing interests

The authors declare that they have no competing interests.

## Authors’ contributions

KGG performed the analyses and wrote the analysis software, assisted in the experiments and wrote the manuscript. EV designed the experiment, wrote the processing software, assisted in data processing and interpretation and writing the manuscript. JG designed the experiment, assisted in data processing and interpretation and writing the manuscript. HHM designed the experiment and critically reviewed the manuscript. DW conducted the experiments and recruited the patients and critically reviewed the manuscript. RWH performed the clinical assessment and critically reviewed the manuscript. JA took part in discussions on the outcome and assisted in writing the manuscript. CM designed the experiment, performed the clinical assessment, assisted in data processing and interpretation and writing the manuscript. All authors read and approved the manuscript.
